# Sexual Harassment Among Medical residents and interns in Sfax: Psychological Impact

**DOI:** 10.1192/j.eurpsy.2025.2288

**Published:** 2025-08-26

**Authors:** S. Ajmi, F. Cherif, O. Bouattour, D. Mnif, I. Feki, R. Masmoudi, J. Masmoudi

**Affiliations:** psychiatry A department, Hedi Chaker hospital university, Sfax, Tunisia

## Abstract

**Introduction:**

Sexual harassment (SH) is a pervasive issue, particularly in workplace environments. The medical field, especially in hospitals, is not immune to this issue with medical residents and interns often being disproportionately affected. SH can have profound psychological, emotional, and physical consequences, which can impair professional performance and well-being.

**Objectives:**

To assess the psychological impact of SH among medical residents and interns working in hospitals in Sfax Tunisia.

**Methods:**

We conducted a cross-sectional and descriptive study involving medical residents and interns working in hospitals in Sfax. Data were collected using an anonymous self-questionnaire. This questionnaire was published on social media during January and February 2024. It included sociodemographic characteristics, medical history, psychoactive substance use, professional data, and experiences related SH. The Depression Anxiety Stress Scale (DASS-21) was used to assess the psychological distress of the participants.

**Results:**

We collected 141 responses, of which 19.9% declined to participate in this study.

Finally, a total of 113 participants, with sex ratio (M/F) of 0.54, were recruited. The average age was 27.92 years. In our population, 20.4% were interns. Among the participants, 68.1% were single, 91.2% were from urban backgrounds.

Among the participants, 41.6% reported experiencing sexual harassment during their practice at the hospitals in Sfax. Verbal harassment was the most common form reported as sexual harassment (43,3%). The assessment of the DASS21 questionnaire showed, that 17 participants had a moderate overall score (15%) and seven participants had a severe overall score (6.2%). In our study, the overall DASS scores (p<0.001), as well as the Depression (p<0.001), Anxiety (p<0.001), and Stress (p=0.002) sub-scores, were significantly higher among participants who were victims of harassment.

**Conclusions:**

The findings underscore the urgent need for implementing preventive measures in hospital settings, providing support for victims, and raising awareness about SH and its consequences.

**Disclosure of Interest:**

None Declared

